# Assessment of Subepidermal Low‐Echogenic Band via High‐Frequency Ultrasound for Evaluating the Efficacy of Platelet‐Rich Plasma Injection in Treating Facial Skin Photoaging: A Case Series

**DOI:** 10.1111/jocd.70684

**Published:** 2026-01-19

**Authors:** Wenjue Ye, Tong Liu, Wei Zhang, Sicheng Peng, Yong Liao

**Affiliations:** ^1^ Beijing Graceline Medical Aesthetic Clinic Beijing China; ^2^ Department of Medicine Bloomage Biotechnology Corporation Limited Beijing China

**Keywords:** dermal thickness, high‐frequency ultrasound, platelet‐rich plasma, skin photoaging, subepidermal low‐echogenic band

## Abstract

**Background:**

Facial skin photoaging manifests as wrinkles, laxity, roughness, enlarged pores, telangiectasia, pigmentation, and dermal structural changes. Histologically, dermal collagen degradation and dermal‐epidermal junction disruption form the subepidermal low‐echogenic band (SLEB) on ultrasound. Platelet‐rich plasma (PRP) injection promotes skin regeneration but lacks objective imaging biomarkers.

**Objective:**

To objectively evaluate the structural efficacy of PRP for facial photoaging by quantifying SLEB width and dermal thickness changes via high‐frequency ultrasound (HFUS), alongside skin analyzer assessments.

**Methods:**

This retrospective case series included 10 patients (Glogau I–III) receiving three monthly PRP injections. The treatment utilized a standardized dual‐step protocol: manual precision injection targeting the deep dermis, followed by mechanical mesogun injection for uniform superficial coverage. SLEB width and dermal thickness were measured at four facial sites using HFUS before and 1 month after treatment. A skin analyzer assessed skin parameters. A comprehensive clinical evaluation was conducted using the Glogau classification and Global Aesthetic Improvement Scale (GAIS) for efficacy and patient satisfaction assessment.

**Results:**

Post‐treatment, HFUS revealed significant reductions in SLEB width (average decrease 29%–47%, all *p* < 0.001) and increases in dermal thickness (average increase 24%–51%, all *p* ≤ 0.003). Skin analyzer showed significant improvements in Pores, Wrinkles, Brown Spots, and Red Areas (all *p* < 0.05). Clinical evaluations confirmed significant improvement in Glogau classification (*p* < 0.01) and high patient satisfaction (100% reported improvement on GAIS). No serious adverse events were observed; only transient erythema/edema and mild pain were reported.

**Conclusion:**

HFUS is a reliable, non‐invasive tool for assessing PRP efficacy. SLEB width reduction serves as a sensitive, objective imaging biomarker, providing structural evidence for PRP's ability to improve photoaging damage.

## 1 Introduction

Skin photoaging refers to structural changes in the skin caused by long‐term ultraviolet exposure. Its key pathological features include degradation of dermal collagen fibers, degeneration of elastic fibers, and structural disruption of the dermal‐epidermal junction (DEJ). In high‐frequency ultrasound imaging, the disruption of the DEJ manifests as a characteristic hypoechoic band, the subepidermal low‐echogenic band (SLEB). Its width is positively correlated with the severity of photoaging and has been confirmed as a sensitive ultrasonic marker reflecting collagen degradation in the papillary dermis, elastosis, and glycosaminoglycan accumulation [1, 2]. Platelet‐rich plasma (PRP) injection, by releasing various growth factors, effectively stimulates fibroblast proliferation and extracellular matrix (ECM) synthesis and has become a commonly used clinical method for skin rejuvenation. A recent bibliometric analysis underscores the sustained and growing research interest in PRP for skin and plastic surgery over the past two decades, highlighting its established role in the field [3]. However, current evaluations of PRP efficacy mostly rely on subjective clinical scores or surface texture analysis, lacking objective imaging indicators that reflect deep skin structural changes. High‐frequency ultrasound (HFUS), as a non‐invasive tool, enables precise quantification of deep structures such as epidermal and dermal thickness and SLEB and has recently shown unique value in monitoring anti‐aging treatments [4, 5]. Recent investigations have further substantiated the utility of ultrasound in tracking the regenerative effects of autologous blood concentrates. Notably, Majewska et al. demonstrated through rigorous ultrasound assessment that standardized PRP and platelet‐rich fibrin (PRF) protocols significantly increased skin density and thickness in the forehead, cheek, and lower eyelid regions, establishing a precedent for sonographic monitoring of these therapies [6]. Similarly, Tejero García et al. utilized HFUS to document the reduction of SLEB width following photothermal bioactivated PRP treatments, reinforcing the link between clinical rejuvenation and measurable ultrasonic structural changes [7]. This case series explores the application value of HFUS, particularly the quantitative measurement of SLEB, in objectively assessing the improvement of structural damage in facial skin photoaging by PRP injection. It also aims to correlate the deep structural improvements revealed by HFUS with apparent clinical scores (Glogau classification) and multidimensional parameters from a skin analyzer to construct a complete evidence chain from microstructure to clinical manifestation.

## 2 Case Series and Methods

### 2.1 Case Data

Retrospectively included were 10 patients with facial photoaging treated between December 2024 and March 2025, aged 28–50 years, with Glogau skin photoaging classification of Grade I–III. Exclusion criteria included: platelet dysfunction or hematologic diseases, receipt of other facial rejuvenation treatments (e.g., laser, radiofrequency, filler injections) within the past 6 months, and pregnant or lactating women. The study protocol conformed to the 1975 Declaration of Helsinki and received the approval of the clinic's institutional review board. All patients provided written informed consent for the use of their photographs.

### 2.2 Treatment Method

#### 2.2.1 PRP Preparation Protocol

Autologous platelet‐rich plasma was prepared using the Regen PRP kit (REGEN LAB SA, Switzerland; approval number: 20173107130, approved by the National Medical Products Administration of China). 8 mL of peripheral venous blood was collected from each patient, and 5 mL of PRP was obtained following centrifugation at 1500× *g* for 9 min using a RegenLab centrifuge (642VFD Plus, Drucker Diagnostics, USA). This system has been reported in previous studies to yield a platelet concentration approximately 1.65–4.4 times that of whole blood [8]. According to the manufacturer's protocol, the prepared PRP yielded a mean platelet concentration approximately 1.6 times higher than that of baseline whole blood.

#### 2.2.2 Standardized Injection Protocol

**FIGURE 1:**
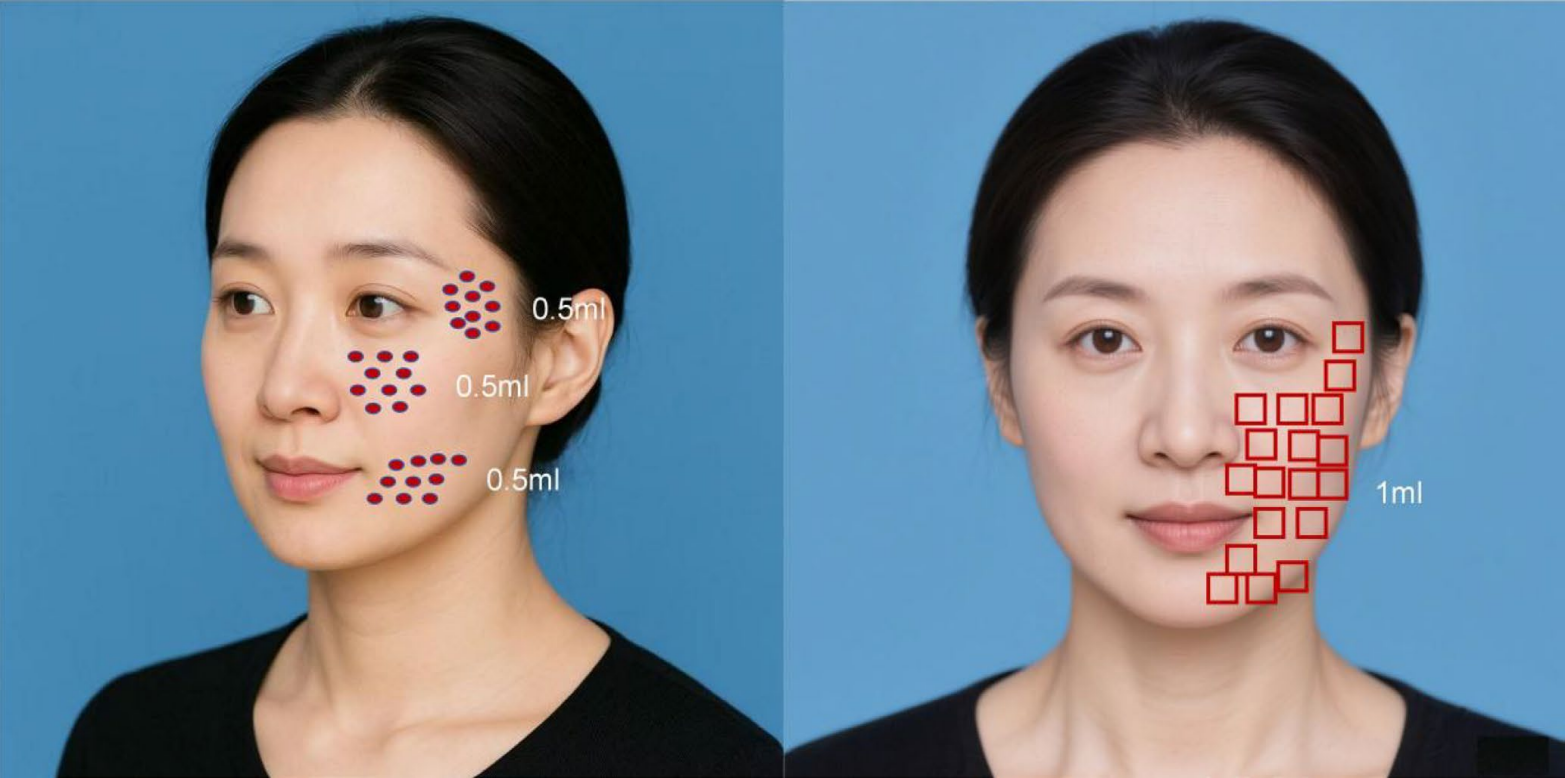
Schematic diagram illustrating the standard injection zones and point distribution. The left panel demonstrates the manual precision injection sites targeting the deep dermis in key areas (temples, infraorbital, nasolabial folds, and perioral area). The right panel shows the mechanical mesogun injection sites for uniform superficial coverage of the facial dermis.

Topical anesthesia (Lidocaine and Prilocaine Cream; Ruyuan East Sunshine Pharmaceutical Co. Ltd., China; NMPA approval number: H20233629) was applied under occlusion for 30–40 min and removed immediately before injection. Using a 34G 1.5 mm needle, 3 mL of PRP was evenly injected into the facial dermis at 0.05 mL per point, spaced 1 cm apart, focusing on key areas including the temples 0.5 mL, infraorbital and nasolabial folds 0.5 mL, and perioral area 0.5 mL. The injection volume was strictly standardized to 1.5 mL per lateral side of the face (3.0 mL in total for the entire face). Then, using a 9‐needle mesogun, 2 mL of PRP was injected into the facial dermis. The device parameters were standardized as follows: single injection dose of 0.0286 mL and suction level 4. A total volume of 1.0 mL of PRP was injected per lateral side (2.0 mL total for the entire face) during this step. Consequently, each patient received a total of 5 mL of PRP per session (3 mL via manual injection and 2 mL via mesogun). Each patient received a total of 3 treatments at 4‐week intervals. A schematic diagram illustrating the standard injection zones and point distribution is provided in Figure 1.

### 2.3 Efficacy Evaluation

The primary evaluation tool was a 14 MHz high‐frequency ultrasound (Portable Color Doppler Ultrasound Diagnostic System, Chengdu Stork Healthcare Co. Ltd., model: cloud‐31). Before the first treatment and 1 month after the final treatment, the same experienced physician measured SLEB width (mm) and dermal thickness (mm) at fixed sites (temporal, infraorbital, nasolabial fold and perioral). To reduce operator‐ and technique‐related variability, ultrasound examinations were performed with standardized settings (fixed gain and depth), in a temperature‐controlled room, with patients in the supine position and a neutral facial expression; measurement sites were pre‐marked using anatomical landmarks and photographed for repeatability. All scans and measurements were performed by the same physician with > 5 years of ultrasound experience. Simultaneously, a skin analyzer (Meiji 3D Skin Analyzer, Hangzhou Xiaofu Technology Co. Ltd., model: AI3D‐220) was used to capture facial images and evaluate Pores, Wrinkles, Brown Spots, and Red Areas indicators. Glogau classification and the Global Aesthetic Improvement Scale (GAIS) were used for clinical overall assessment.

### 2.4 Statistical Analysis

Data analysis was performed using SPSS 26.0 software. Measurement data are presented as improvement degree and mean ± standard deviation. Comparisons before and after treatment were conducted using paired *t*‐tests and Wilcoxon signed‐rank tests. *p* < 0.05 was considered statistically significant. To further quantify the magnitude of the treatment effect, within‐subject effect sizes (Cohen's *d*_*z*_) were calculated for the primary ultrasound outcomes (SLEB width and dermal thickness).

## 3 Results

### 3.1 High‐Frequency Ultrasound Measurement Results

**FIGURE 2:**
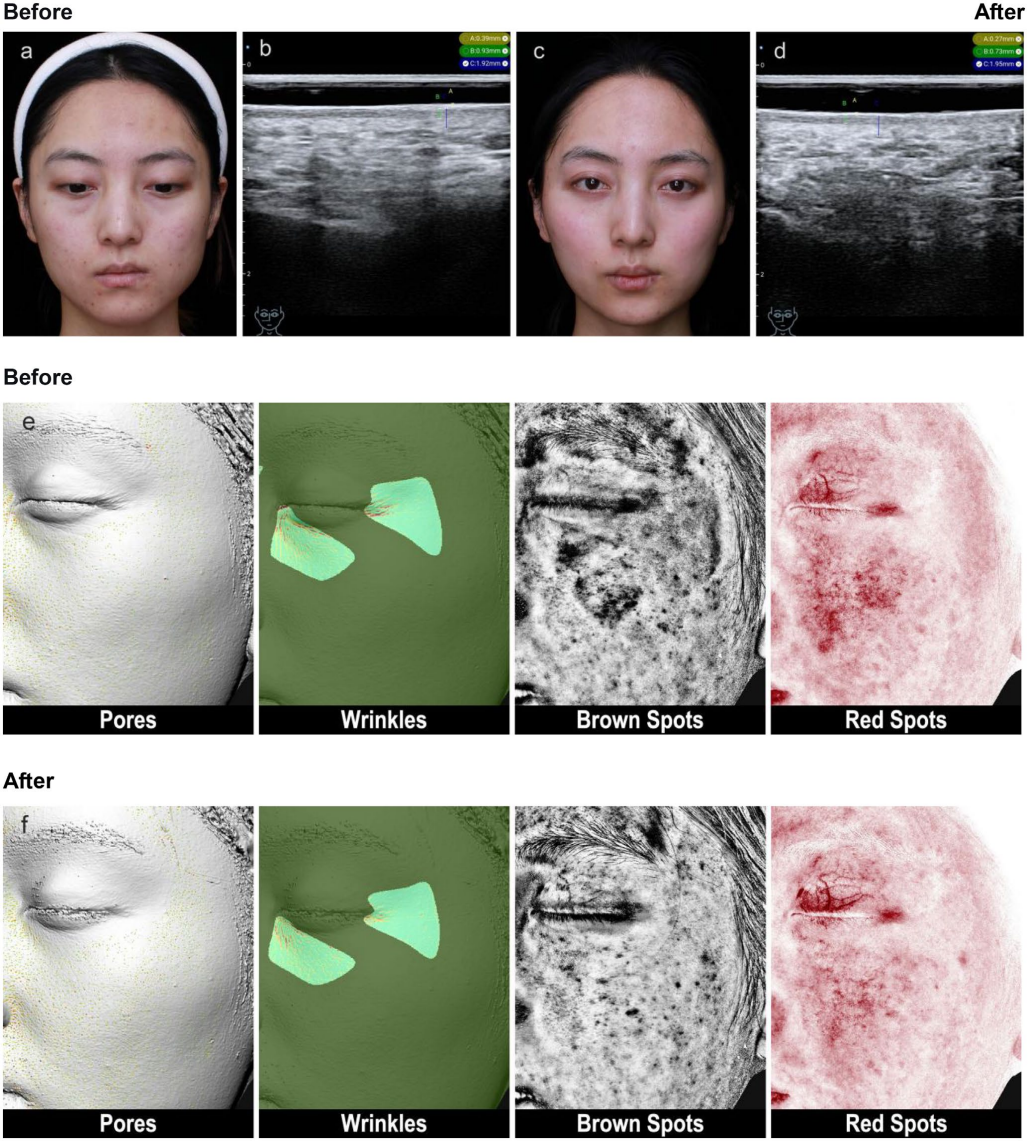
Typical case 1. “26‐year‐old female, relatively dull and sallow complexion, slight redness in the mid‐face apple muscle area, thin stratum corneum, some fine lines under the eyes.” (a) “Clinical image before PRP treatment.” (b) “High‐frequency ultrasound (HFUS) measurement of epidermis, SLEB, and dermal thickness in the nasolabial fold area.” (c) “Clinical image four weeks after the third PRP treatment.” (d) “HFUS measurement of skin thickness in the nasolabial fold area four weeks after the third PRP treatment.” (e, f) “Skin analyzer images of Pores, Wrinkles, Brown Spots, Red Spots before and four weeks after the third PRP treatment.”

**FIGURE 3:**
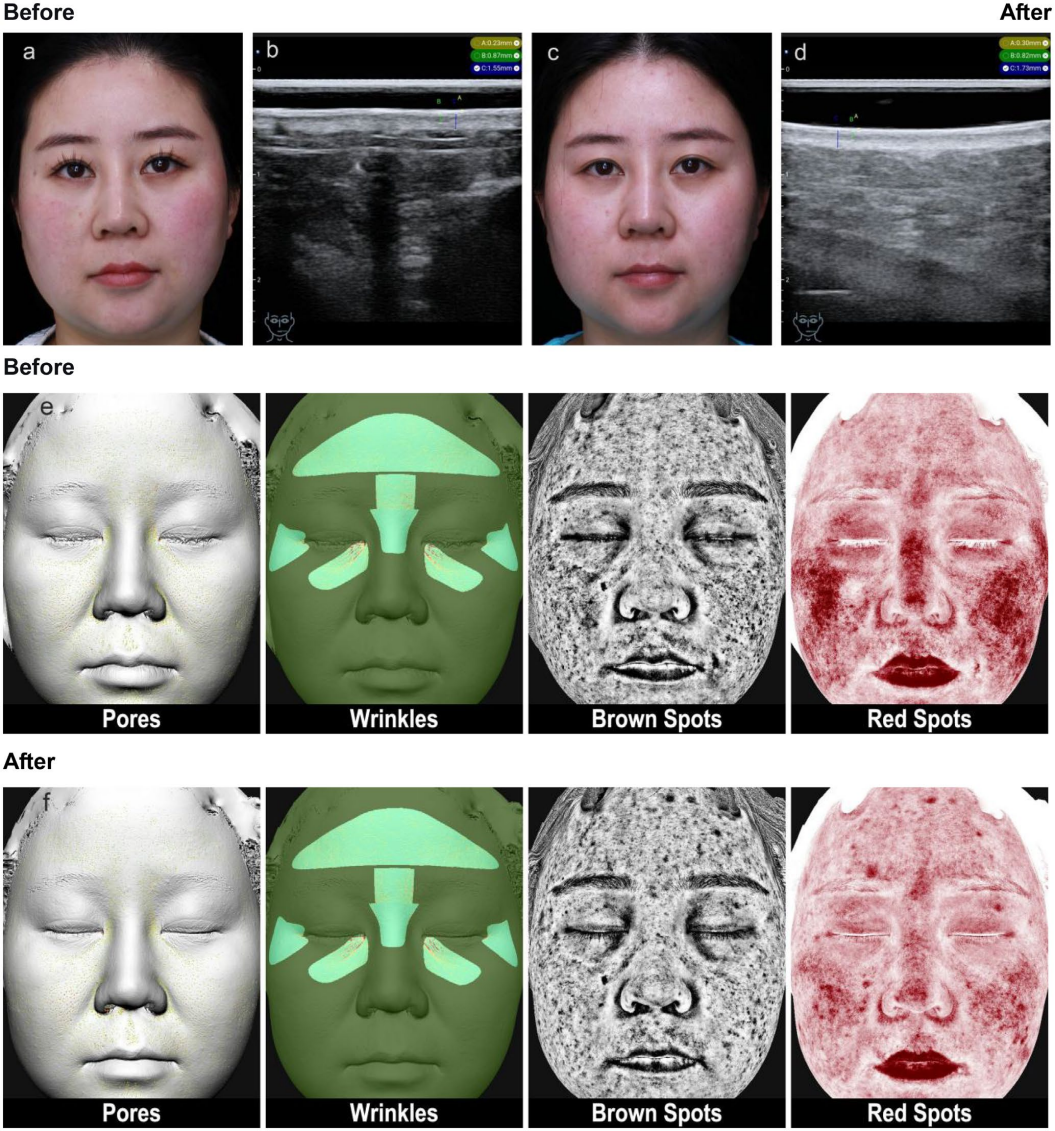
Typical case 2. “33‐year‐old female, facial sensitive skin, redness, thin stratum corneum, mild wrinkles around the eyes.” (a) “Clinical image before PRP treatment.” (b) “High‐frequency ultrasound (HFUS) measurement of epidermis, SLEB, and dermal thickness in the perioral area.” (c) “Clinical image four weeks after the third PRP treatment.” (d) “HFUS measurement of skin thickness in the perioral area four weeks after the third PRP treatment.” (e, f) “Skin analyzer images of Pores, Wrinkles, Brown Spots, Red Spots before and four weeks after the third PRP treatment.”

**FIGURE 4:**
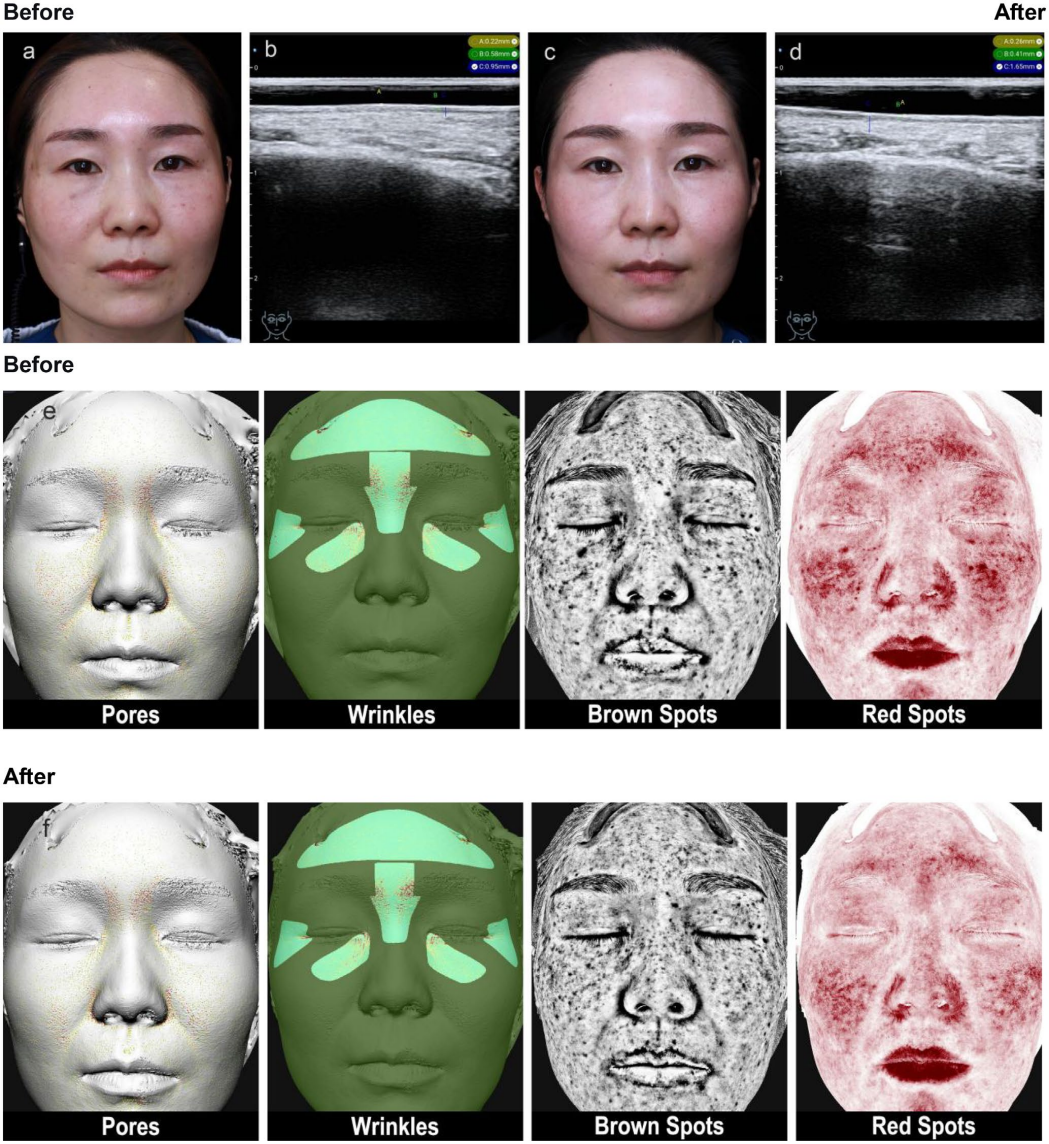
Typical case 3. “38‐year‐old female, sensitive and red skin, thin skin, dynamic wrinkles around the eyes.” (a) “Clinical image before PRP treatment.” (b) “High‐frequency ultrasound (HFUS) measurement of epidermis, SLEB, and dermal thickness in the infraorbital area.” (c) “Clinical image four weeks after the third PRP treatment.” (d) “HFUS measurement of skin thickness in the infraorbital area four weeks after the third PRP treatment.” (e, f) “Skin analyzer images of Pores, Wrinkles, Brown Spots (superficial pigmentation, deep pigmentation, spot area), Red Spots before and four weeks after the third PRP treatment.”

All 10 patients completed the treatment and follow‐up. HFUS measurements showed that after PRP injection treatment, SLEB width significantly decreased in all four facial regions (*p* < 0.001), with average reduction amplitudes of 47% in the infraorbital area, 46% in the temporal area, 40% in the nasolabial fold area, and 34% in the perioral area. The effect sizes indicated a very large treatment effect across all areas: Infraorbital (Cohen's *d*_*z*_ = 4.74), Temporal (Cohen's *d*_*z*_ = 2.97), Nasolabial (Cohen's *d*_*z*_ = 2.85), and Perioral (Cohen's *d*_*z*_ = 1.89). Concurrently, dermal thickness significantly increased in all regions (*p* ≤ 0.003), with average increase amplitudes of 51% in the infraorbital area, 33% in the temporal area, 32% in the nasolabial fold area, and 24% in the perioral area. Dermal thickness increase again showing strong effect sizes: Infraorbital (Cohen's *d*_*z*_ = 1.57), Temporal (Cohen's *d*_*z*_ = 2.45), Nasolabial (Cohen's *d*_*z*_ = 1.33), and Perioral (Cohen's *d*_*z*_ = 1.28) (details in Table 1). Ultrasound images of typical cases (Figures 2, 3, 4) showed thinning of the SLEB and enhanced dermal echogenicity after treatment, suggesting improved collagen density.

**TABLE 1:** HFUS measurement results before and after treatment (*n* = 10).

Case no.	SLEB width improvement				Dermal thickness improvement			
	Temporal	Infraorbital	Nasolabial	Perioral	Temporal	Infraorbital	Nasolabial	Perioral
1	−35%	−45%	−39%	−16%	27%	34%	89%	67%
2	−29%	−29%	−29%	−40%	44%	47%	12%	24%
3	−42%	−43%	−22%	−48%	22%	56%	27%	22%
4	−53%	−37%	−28%	−46%	24%	7%	26%	7%
5	−51%	−56%	−53%	−9%	22%	17%	34%	16%
6	−81%	−63%	−62%	−66%	38%	70%	27%	27%
7	−46%	−45%	−31%	−30%	14%	16%	14%	12%
8	−34%	−54%	−31%	−43%	33%	102%	21%	21%
9	−33%	−52%	−58%	−11%	48%	88%	53%	45%
10	−58%	−43%	−45%	−34%	56%	71%	12%	3%
Mean ± SD	−46% ± 15%	−47% ± 10%	−40% ± 13%	−34% ± 19%	33% ± 14%	51% ± 31%	32% ± 25%	24% ± 19%
*p*‐value	< 0.001	< 0.001	< 0.001	< 0.001	< 0.001	< 0.001	0.003	0.003
Cohen's *d*_*z*_	−2.97	−4.74	−2.85	−1.89	+2.45	+1.57	+1.33	+1.28

### 3.2 Four‐Dimensional Skin Photoaging Assessment Results

The skin analyzer showed significant improvements in Pores, Wrinkles, Brown Spots, and Red Areas after treatment (*p* < 0.05), with improvement degrees of 2.18 ± 3.36, 3.75 ± 4.43, 4.26 ± 1.66, and 5.49 ± 5.02, respectively (details in Table 2).

**TABLE 2:** Comparison of skin analyzer results and Glogau classification before and after treatment (*n* = 10) $\left(\bar{X}\pm S\right)$.

Parameter	Before treatment $\left(\bar{X}\pm S\right)$	After treatment $\left(\bar{X}\pm S\right)$	Paired *t*‐test *p*‐value	Median improvement [IQR]	Wilcoxon test *p*‐value
Continuous normal data					
Pores	80.05 ± 6.86	81.50 ± 4.71	0.049	—	—
Wrinkles	74.25 ± 8.42	78.95 ± 10.26	0.025	—	—
Brown Spots	74.14 ± 8.76	78.46 ± 9.22	< 0.001	—	—
Red Areas	70.73 ± 20.13	75.61 ± 20.83	0.007	—	—
Continuous non‐normal/Ordinal data					
Glogau skin aging classification	2.10 ± 0.32	1.10 ± 0.32	—	1.0 [1.0, 1.0]	0.002

*Note:* Continuous variable data were tested for normality. The improvement values for Pores, Wrinkles, Brown Spots, and Red Areas conformed to a normal distribution; therefore, paired *t*‐tests were used, and data are presented as mean ± standard deviation. Glogau skin aging classification is an ordinal categorical variable; therefore, the Wilcoxon signed‐rank test was used, and data are presented as median [interquartile range]. For the skin analyzer parameters (Pores, Wrinkles, Brown Spots, Red Areas), higher scores indicate better skin condition; thus, increases represent improvement.

### 3.3 Clinical Overall Assessment Results and Safety

According to the investigator's assessment, the improvement in Glogau skin aging classification in the treatment group was highly statistically significant (*p* < 0.01) and demonstrated a satisfactory clinical outcome (100% response rate). According to the subjects' assessment, 100% of subjects in the treatment group self‐reported positive improvement on the GAIS, with 70% feeling “very much improved” and 30% feeling “much improved”. No subjects reported “no change” or “worse” (details in Table 2).

Regarding safety, throughout the treatment course and the one‐month follow‐up period, none of the 10 patients reported any serious local or systemic adverse events. A minority of patients (*n* = 3) experienced expected, mild, and transient injection‐site reactions, including erythema, mild swelling, and bruising, which resolved spontaneously within 24–72 h. No infections, allergic reactions, nodule formation, or other complications were observed.

## 4 Discussion

As noted by Shasha Zhao et al., facial rejuvenation treatments are diverse, but there is currently a lack of a standardized efficacy evaluation system, making it difficult to compare the effects of different treatment modalities [9, 10]. This study is the first to systematically use quantitative changes in SLEB under HFUS as a core indicator to evaluate the structural efficacy of PRP in treating facial photoaging. The results show that the significant reduction in SLEB width (average 42%) and the significant increase in dermal thickness (average 35%) are highly consistent, providing strong objective imaging evidence that PRP may assist in repairing the DEJ structural damage and dermal matrix loss caused by photoaging. It is noteworthy that in individual cases, extremely pronounced improvements were observed (e.g., Case 6 showed an 81% reduction in Temporal SLEB; Case 8 showed a 102% increase in Infraorbital dermal thickness). These large percentage changes, while outliers relative to the mean, likely reflect specific individual responsiveness to growth factors or potentially lower baseline values that magnify percentage calculations.

Through a multi‐assessment system, this study objectively confirmed the significant improvement effect of PRP injection on facial photoaging. Clinically, patients' Glogau classification improved from an average of 2.10 to 1.10 after treatment. This reversal specifically manifested as the fading of dynamic wrinkles caused by muscle contraction, refinement of skin texture, and improvement in pigmentation uniformity, aligning with those reported by Elnehrawy et al. regarding the effectiveness of PRP in improving various types of wrinkles [11]. Simultaneously, the objective data from the skin analyzer further corroborated the above clinical observations, with significant improvements (*p* < 0.05) in Brown Spots, Red Spots, Wrinkles, and Pores, revealing the optimization of the skin's apparent state from a quantitative perspective. In previous studies, including all three split‐face randomized controlled trials, the clinical efficacy of PRP injection has been reported, specifically manifested as: increased epidermal and papillary dermal thickness, increased collagen volume and fibroblast numbers, reduced dark circles, improved wrinkles, improved skin evenness and texture, improved crow's feet, reduced number of brown spots, and overall aesthetic improvement [12, 13, 14]. The known multiple effects of PRP—promoting keratinocyte proliferation, repairing skin barrier function, anti‐inflammatory effects, and regulating angiogenesis [15, 16, 17], collectively form the physiological basis explaining the aforementioned spot fading and red area improvement. These cellular activities are directly supported by in vitro evidence demonstrating that PRP stimulates the proliferation and migration of human dermal fibroblasts [18], with certain formulations like fluid platelet‐rich fibrin showing even greater efficacy in promoting fibroblast migration, proliferation, and collagen synthesis compared to traditional PRP [19]. These findings are consistent with previous studies indicating that PRP acts by stimulating fibroblast proliferation, increasing anti‐inflammatory factors, and producing angiogenic factors and extracellular matrix remodeling proteins (including type I procollagen, hyaluronic acid, and tissue inhibitors of metalloproteinases) [20, 21]. This leads to increased collagen remodeling, epidermal thickening, increased vascularization, and reduced degradation of the dermal extracellular matrix, all essential for skin rejuvenation [22].

This study observed an average reduction in SLEB width of 42% after treatment, alongside an average increase in dermal thickness of 35%. SLEB, as a characteristic marker of photoaged skin under ultrasound, histologically represents flattening of the dermal‐epidermal junction (DEJ), damage to the basement membrane zone, and collagen degradation and abnormal elastic fiber deposition in the papillary dermis [1, 2, 3, 4, 5, 23, 24]. Therefore, the significant thinning of SLEB strongly suggests that the growth factors abundant in PRP (e.g., TGF‐β, PDGF, VEGF) effectively activated fibroblasts, thereby promoting the regeneration and orderly remodeling of basement membrane components such as type IV collagen and anchoring fibrils, as well as type I and III dermal collagen [25, 26, 27, 28]. This “inside‐out” structural repair is the fundamental reason for the observed clinical improvement in Glogau classification and skin quality. These findings are consistent with Díaz et al.'s report of enhanced dermal echogenicity (suggesting increased collagen density) after PRP treatment [29]—a conclusion further strengthened by recent high‐level clinical evidence from a split‐face trial confirming PRP's efficacy in stimulating dermal remodeling [30]—and we further focused the observation on SLEB, a target with greater pathological specificity [31]. This aligns perfectly with the view of Vergilio et al. that “improvement in dermal collagen and organizational structure is the cornerstone of clinical rejuvenation” [1], and also confirms the potential value proposed by Pequeno ALV et al. of using SLEB as a sensitive indicator for monitoring photoaging treatment [32]. Concurrently, we also found that PRP treatment showed relatively prominent effects in improving SLEB and skin thickness in the infraorbital area: on average, the infraorbital area showed the numerically largest improvement amplitudes in both indicators (greatest SLEB reduction at 47%, greatest dermal thickening at 51%). Although the differences between regions did not reach statistical significance (*p* > 0.05), the observed effect sizes were large, and the numerical trend suggests the greatest improvement in the infraorbital area. This result suggests possible regional differences, requiring further validation with larger sample sizes, and this finding is consistent with the results of Majewska L's study, which showed significant improvement in skin density and thickness via ultrasound and statistical analysis, particularly in the lower eyelid area [6, 33, 34]. Similarly, Tejero García et al. observed a reduction in SLEB width following photothermal bioactivated PRP treatment for hand rejuvenation [7]. Furthermore, a dedicated clinical study on facial rejuvenation with pure PRP has documented significant reductions in SLEB thickness, corroborating our primary finding [35]. The consistency of our SLEB reduction data with these independent studies reinforces the validity of SLEB as a responsive biomarker for PRP efficacy.

Another important finding is the great potential of HFUS as an early and objective predictive indicator [36, 37]. Among all evaluation parameters, the reduction in SLEB width measured by HFUS was the indicator with the largest magnitude of change and the strongest statistical significance. Particularly noteworthy is that when apparent parameters such as “Wrinkles” measured by the skin analyzer had not yet reached extremely significant improvement, HFUS had already clearly captured the significant optimization of deep structures—SLEB thinning and dermal thickening. Effect size analysis further revealed that the magnitude of improvement in SLEB width (average effect size 3.0) was significantly greater than the increase in dermal thickness (average 1.5); this statistical characteristic strongly supports the argument that SLEB serves as a more sensitive early biomarker. This “dissociation phenomenon” has important clinical implications: HFUS can sensitively detect positive biological remodeling of the dermal matrix earlier than clinically visible apparent changes.

The limitations of this study include the small sample size (*n* = 10) and lack of a control group. Consequently, the observed dermal structural improvements cannot be completely isolated from the potential contribution of the injection procedure itself (microtrauma and mechanical stimulation). Given the exploratory nature of this study, *p*‐values for multiple anatomical sites were not corrected for multiplicity, increasing the risk of Type I errors. Additionally, the use of a 14 MHz probe, while practical, limits the precision of absolute SLEB thickness measurement compared to higher frequency probes (≥ 20 MHz or even 30–50 MHz). But the relative changes remain a valid clinical indicator. Future prospective randomized controlled studies with larger samples and extended follow‐up periods (at 3 and 6 months) are needed to observe the durability of the efficacy. Furthermore, dermatologic ultrasound is operator‐dependent; although we standardized settings and used a single experienced operator, external reproducibility should be confirmed in multi‐operator studies.

## 5 Conclusion

High‐frequency ultrasound is an effective, non‐invasive assessment tool. This study provides objective imaging evidence suggesting that PRP injection can significantly improve the microstructure of facial photoaged skin, specifically manifested as narrowing of SLEB width and an increase in dermal thickness. More importantly, through correlation analysis, this study confirms that these deep structural improvements observed by HFUS, together with the improvement in Glogau clinical classification and the optimization of apparent parameters in multiple dimensions (Wrinkles, Pores, Brown Spots, Red Areas) from the skin analyzer, form a mutually supportive and corroborating evidence chain. Among these, SLEB can serve as a sensitive and objective imaging biomarker for evaluating the efficacy of PRP, providing a reliable basis for clinical efficacy judgment.

We recommend that in future clinical research and practice concerning skin regeneration therapies, quantitative measurement of SLEB under high‐frequency ultrasound should be incorporated as a key objective assessment indicator, combined with traditional clinical scores and skin surface analysis, to construct a more comprehensive and multi‐dimensional efficacy evaluation system.

## Author Contributions

W.Y. and Y.L. conceived and designed the study; T.L. collected the data and performed the statistical analysis; Ultrasound examinations were performed and interpreted by W.Y. and W.Z.; Injecting procedures were carried out by W.Y., W.Z., and S.P.; The manuscript was written by T.L. and critically revised by W.Y. and Y.L. All authors reviewed and approved the final version of the manuscript.

## Ethics Statement

The study protocol conformed to the 1975 Declaration of Helsinki and received the approval of the clinic's institutional review board. All patients provided written informed consent for the use of their photographs (Institutional Review Board of Beijing Graceline Medical Aesthetic Clinic; IRB No. 2025012).

## Conflicts of Interest

The authors declare no conflicts of interest.

## Data Availability

The data that support the findings of this study are available on request from the corresponding author. The data are not publicly available due to privacy or ethical restrictions.
